# Sharks and Rays of Northern Australia’s Roper River, with a Range Extension for the Threatened Speartooth Shark *Glyphis glyphis*

**DOI:** 10.3390/ani14223306

**Published:** 2024-11-17

**Authors:** Julia M. Constance, Erica A. Garcia, Yugul Mangi Rangers, Christy-Louise Davies, Peter M. Kyne

**Affiliations:** 1 Research Institute for the Environment and Livelihoods (RIEL), Charles Darwin University, Ellengowan Drive, Darwin, NT 0810, Australia; 2 Yugul Mangi Rangers, Northern Land Council, Ngukurr, NT 0852, Australia; 3 Ngururrpa Ranger Program, Balgo, WA 6770, Australia; 4 Desert Support Services, Perth, WA 6005, Australia

**Keywords:** elasmobranchs, conservation, threatened species, euryhaline species, sawfish

## Abstract

Northern Australia is considered a ‘lifeboat’ region for globally threatened shark and ray species (elasmobranchs), although much of the region is understudied. The Roper River in the Northern Territory’s Gulf of Carpentaria has been inadequately surveyed, with most elasmobranch data gained opportunistically through freshwater fish surveys. This study aimed to report the occurrence of elasmobranch species in the Roper River through targeted field surveys conducted between 2016 and 2024 and to review data from other sources. Four euryhaline species were recorded, comprising two sharks, a sawfish, and a stingray. Records of the globally Vulnerable speartooth shark (*Glyphis glyphis*) represented a significant range extension and the documentation of a new reproductive population. Records of the Critically Endangered largetooth sawfish (*Pristis pristis*) extended almost 400 km upstream, highlighting extensive use of the river system. A predominantly marine species, the Critically Endangered giant guitarfish (*Glaucostegus typus*) was observed in brackish estuarine waters, approximately 15 km upstream from the river mouth. Further research is required to develop a more thorough understanding of abundance, life history, and population connectivity for these species in this river system. Given the documentation of threatened species, the potential impacts of fisheries, water allocations, and climate change require assessment.

## Simple Summary

Northern Australia represents an important region for the conservation of globally threatened sharks and rays; however, much of the region has been understudied. We aimed to survey the Roper River system of the Northern Territory of Australia for sharks and rays and compile records from other sources. Five shark and ray species were recorded, including four euryhaline species which can occur in any level of salinity, and one Critically Endangered marine species. This study found that the Roper River is a reproductive area for the observed euryhaline species and is used extensively, with some species extending almost 400 km upstream. Further research is required to understand the abundance and biology of sharks and rays in the Roper River, and how they may respond to human-driven threats.

## 1. Introduction

Understanding the distributions of animals in space and over time has become increasingly important to evaluate ecological processes, to understand how species may respond to anthropogenic threats, including climate change, and to measure the impact of conservation efforts [1]. Knowledge gaps in geographic ranges and the environmental drivers of distributions of aquatic species have limited the implementation of appropriate ecosystem-based management [2]. Understanding the distribution of species throughout their various life stages and across habitats is essential for effective conservation planning and the protection of habitats [3,4]. Area-based management is widely being implemented to protect biodiversity in terrestrial, freshwater, and marine environments, with significant commitment to implementing protected areas from the international community [5]. Area-based management is also increasingly being utilised to maximise conservation outcomes for elasmobranchs (sharks and rays), and can incorporate biological, behavioural, and ecological characteristics of species [4].

The conservation of elasmobranchs is a global priority. One-third of all species (33.3%; 397 species) are at risk of extinction (Vulnerable, Endangered, Critically Endangered), and a further 13.7% (163 species) are categorised as Data Deficient according to the International Union for the Conservation of Nature (IUCN) Red List of Threatened Species [6]. Elasmobranch species which utilise non-marine environments such as estuaries and rivers during critical stages of their life histories are particularly at risk of extinction. These animals have an elevated risk of exposure to anthropogenic pressures such as overfishing, habitat loss and degradation, and pollution [7,8]. Euryhaline generalist (hereafter, ‘euryhaline’) elasmobranchs are a group of ten unique species which are physiologically capable of occurring throughout salinity gradients ranging from marine (~35 ppt) to freshwater (<5 ppt) [8]. These species generally use freshwater and/or estuarine environments for particular life stages (e.g., nursery areas) [8]. Significant gaps in knowledge exist for euryhaline elasmobranchs, with most species lacking key life history, movement ecology, and habitat use data [9].

Northern Australia is considered a ‘lifeboat’ region for globally threatened elasmobranchs primarily due to its low human population density [10,11,12]. The region is home to half (five species) of the world’s known euryhaline elasmobranchs [8], four of which are threatened globally [6]. The region still has vast swathes of uninhabited land, intact wetlands and free-flowing river systems, as well as Marine Protected Areas (MPAs) and areas closed to commercial fishing [13,14,15,16]. Due to its remoteness, limited access to many areas, and the costs and logistics associated with research, northern Australian waters have been studied relatively little, with many areas lacking any dedicated elasmobranch surveys. Euryhaline elasmobranchs in particular were, until recently, often only recorded opportunistically during freshwater fish surveys, e.g., [17,18,19]. Northern Australia is under increasing development pressure, and the extraction of groundwater or the ‘harvesting’ of surface water during wet season flow events is increasingly being utilised for irrigation and resource extraction [20].

Although distributional data for euryhaline elasmobranchs in remote regions of Australia has improved significantly over the last two decades, e.g., [21,22,23,24], many areas have yet to be surveyed. One such example of limited survey effort is the Roper River of Australia’s Northern Territory (NT). This river has had one targeted elasmobranch survey, with sampling effort focused on freshwater upstream environments, with only two downstream sampling sites close to the river mouth [25]. Given the lack of data on the distribution, biology, and habitat requirements of elasmobranchs in many remote river systems [9], this study aims to determine the occurrence of elasmobranchs in the Roper River through species-specific targeted surveys and a review of the literature and other data sources.

## 2. Materials and Methods

### 2.1. Study Site

**Figure 1:**
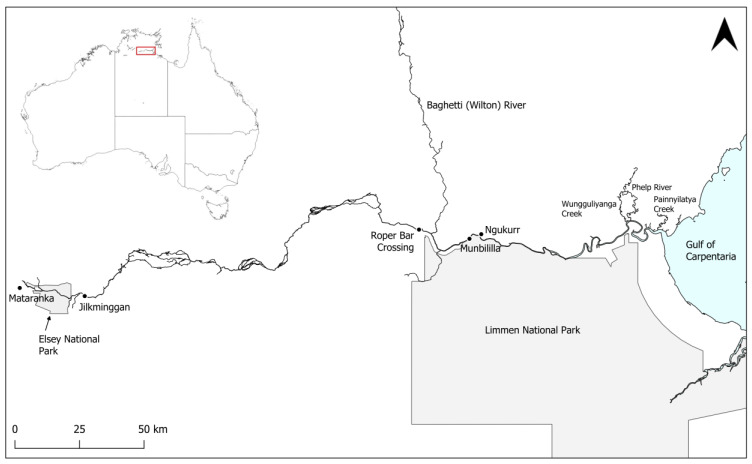
The Roper River in the south-west Gulf of Carpentaria, Northern Territory, Australia.

The Roper River is a large river system in the south-west Gulf of Carpentaria in the wet–dry tropics of the Northern Territory, Australia (Figure 1). Its catchment of almost 80,000 km^2^ makes it one of the largest systems in northern Australia [26,27]. Water flow is seasonally variable, with most (>90%) occurring during the monsoonal rainfall of the wet season (roughly, November–April) [20,26]. Over half (56%) of the Roper River’s streamflow originates from its primary tributaries, which are the Baghetti (Wilton) and Hodgson Rivers, as well as coastal floodplains [27]. The Roper River is, however, a permanently flowing (perennial) tropical river, fed in the dry season by groundwater discharge from the regional Cambrian Limestone Aquifer (CLA) driving flow for over 200 km upstream of the tidal limit [20,27]. The lower Roper River has tidal influence reaching Roper Bar (150 km upstream) [26]. Flow is unregulated, with no dams or weirs [27]. The lower 100 km of the river is vegetated with mangroves, while freshwater riparian plant species such as *Melaleuca* spp., *Eucalyptus camaldulensis*, and *Casuarina cunninghamiana* occur upstream from around 67 km from the mouth [28]. The lower Roper River has a muddy/sandy substrate, while the upper reaches are primarily rocky and sandy.

The human population in the Roper River catchment is very small, at approximately 2500 people [27]. The predominant land use is cattle grazing, occupying 46% of the catchment land [26,27]. Aboriginal freehold tenure accounts for 45% of the catchment, with the entire area to the north of the Roper River making up the South-East Arnhem Land Indigenous Protected Area [27,29]. National parks account for 6% of the catchment land, followed by irrigated horticulture (0.02%) and mining (<0.01%) [26,27]. Existing CLA groundwater use entitlements amount to 33 GL/year, and currently there is limited existing surface water extraction (0.1 GL) [27]. There is significant interest in markedly increasing the extraction of water for the development of agriculture in the region [27]. Recreational and subsistence fishing occurs throughout the Roper River, with commercial fishing concentrated at the mouth of Port Roper and the surrounding coastal waters [30]. The Roper River was closed to commercial net fishing in 1991 due to the potential impact on recreational fishing and tourism [26]. The river is popular among recreational fishers, with most effort primarily occurring in the upstream stretches near Munbililla (Tomato Island) and the mouth and coastal waters.

### 2.2. Elasmobranch Surveys

Targeted threatened elasmobranch surveys were conducted in October–November 2016 (3 days) and October 2017 (4 days) for largetooth sawfish *Pristis pristis*, and September 2023 (5 days), November 2023 (7 days), and October 2024 (6 days) for speartooth shark *Glyphis glyphis*. Data for all elasmobranch species encountered during the surveys were collected. Surveys were conducted from the mouth of the Roper River to just upstream of the Roper Bar crossing, including some tributaries, such as the Baghetti (Wilton) and Phelp Rivers and Wungguliyanga and Painnyilatya Creeks. Survey site selection was based on habitat suitability for the target species, with freshwater reaches and pools targeted for *P. pristis* and turbid brackish tidal reaches for *G. glyphis*. As surveys were targeted towards *P. pristis* and *G. glyphis*, survey site selection may have resulted in lower catch of other species.

All survey sites were tidally influenced to varying degrees, although this was minimal in upstream reaches. Sampling was conducted using 6-inch monofilament gillnets (29 m or 58 m length) and baited hook-and-line (rod or hand line). Circle hooks of size 5/0 to 9/0 were used and bait consisted of fresh-caught bony fishes local to the area (e.g., bony bream *Nematalosa erebi*, barramundi *Lates calcarifer*, popeye mullet *Rhinomugil nasutus*).

Each captured elasmobranch was measured using a 3 m measuring tape (total length [TL] for sharks and sawfish; disc width [DW] for stingrays), sexed, assessed for maturity, sampled for future genetic analysis, photographed, and tagged with a PIT tag to assess recaptures. Additionally, freshwater whiprays (*Urogymnus dalyensis*) were weighed using a vinyl sling and hanging scales rated to 100 kg. Life stage and maturity were assessed based on the presence/absence of an umbilical scar, whether the umbilical scar was open or closed, the calcification of claspers for males, and by observation of pregnancy for females (i.e., clearly distended abdomen). Neonates were defined as animals less than approximately one month old with visible open or closed umbilical scars [31]. Juveniles were defined as individuals older than one month (with no visible umbilical scar) but not yet approaching maturity. Subadults were defined as individuals approaching maturity, with subadult males having partially calcified claspers. The maturity of females could not be assessed externally (aside from mature pregnant females); therefore, size classes of males (which generally mature at a smaller size than females) and predicted sizes at maturity were used to estimate female maturity. All animals were released after processing at the site of capture.

Environmental variables (water temperature, water depth, salinity [in Practical Salinity Scale; PSS], and turbidity [in New Turbidity Units; NTU]) were recorded at each survey site.

Differences in size between males and females were tested with two-sample *t*-tests with significance set at *p* < 0.05. Catch-per-unit-effort (CPUE) was calculated as the number of individuals caught per hook per hour for baited hook-and-line fishing, and as the number of individuals caught per 100 m net length per hour for gillnet fishing. CPUE was calculated for target species only (*G. glyphis* and *P. pristis*).

### 2.3. Literature and Data Review

**Figure 2:**
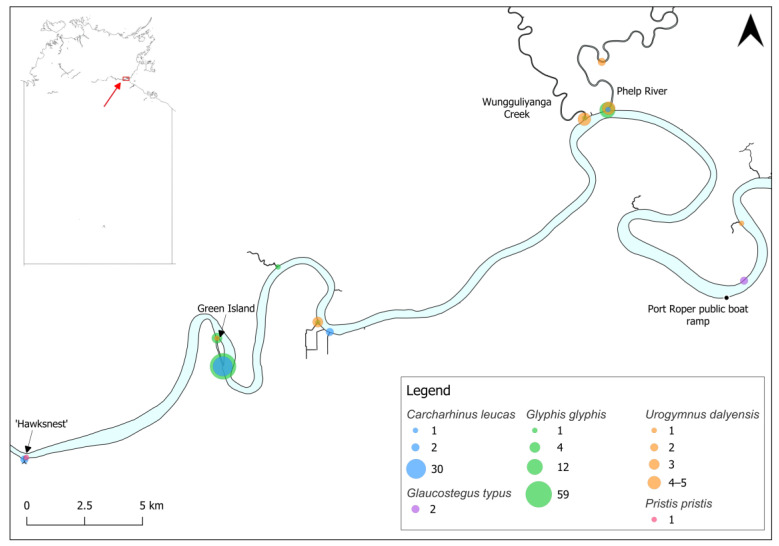
Species records in the lower Roper River, Northern Territory, Australia. Size of circles indicates the number of animals caught or observed (in the case of *G. typus* and *P. pristis*) in each location (refer to legend).

Literature and data sources were reviewed for additional records of elasmobranchs in the Roper River. To ensure records were primarily of euryhaline elasmobranchs, records were collected from the estuary (approximately 6 km downstream of the Port Roper public boat ramp; Figure 2) to the upstream extent of the river and its tributaries, excluding marine waters. The review used the following approach: (1) the terms ‘Roper River’ AND ‘shark’, ‘ray’, or ‘sawfish’ were searched on Google Scholar; (2) the online spatial databases Atlas of Living Australia [32] and iNaturalist [33] were searched for each northern Australian euryhaline elasmobranch (bull shark *Carcharhinus leucas*, northern river shark *Glyphis garricki*, *G. glyphis*, *P. pristis*, *U. dalyensis*); and (3) public records were sourced from data contributed to the Northern Territory Fisheries sawfish database [34]. These records were supplied by recreational fishers and other members of the public.

## 3. Results

Across the 2016, 2017, 2023, and 2024 targeted surveys, 162 individual elasmobranchs were captured via gillnet or baited hook-and-line methods (handline or rod). Gillnets were deployed for 240 × 100 m net hours and caught 28 elasmobranchs. One hundred and thirty-four animals were captured over 534.8 hook hours. Elasmobranchs comprised four euryhaline species from three families: *Carcharhinus leucas* (Carcharhinidae, n = 69), *Glyphis glyphis* (Carcharhinidae, n = 78), *Pristis pristis* (Pristidae, n = 1), and *Urogymnus dalyensis* (Dasyatidae, n = 14). Two *C. leucas* were recaptured the day after their initial capture in the same location. Additional observations were made of one marine species: *Glaucostegus typus* (Glaucostegidae, n = 2). Elasmobranchs were captured at depths of 0.8–9.5 m, water temperatures of 27.0–31.4 °C, turbidity of 3.2–549.0 NTU, and in fresh (salinity 0.06 PSS) to brackish water (23.6 PSS). The literature and data review produced further records of *C. leucas*, *P. pristis*, and *U. dalyensis*, but did not produce records of *G. garricki* or *G. glyphis* (Table 1).

### 3.1. Carcharhinus leucas

Sixty-nine neonate (n = 28) and immature (n = 41) *C. leucas* were captured from 2016 to 2024 (gillnet, n = 27; hook-and-line, n = 42). Sharks ranged 67.5–139.5 cm TL in size, with an average of 83.4 ± 16.0 cm TL. The sex ratio was 1:1.16 (F:M), with no significant difference between the size of males and females (two-sample *t*-test, *p* = 0.53). Twenty-eight neonates were captured during October and November 2016 and October 2024 with a TL of 67.5–86.0 cm. *Carcharhinus leucas* were captured in very shallow (0.8–4.9 m depth) water with a salinity of 0.06–11.9 PSS, turbidity of 3.2–549.0 NTU, and water temperatures of 27.9–31.4 °C.

The literature and data review produced an additional fourteen *C. leucas* caught in freshwater by gillnet, hook-and-line, or long-line methods, or observed swimming, although sizes were not reported for these [18,25,32]. Individuals caught in 2002 (n = 12) were in salinities of 0.3–0.7 ppt, Secchi depths (water transparency) of 80–130 cm, depths of 3.5–21.0 m, and water temperatures of 23.1–24.3 °C [25].

**Figure 3:**
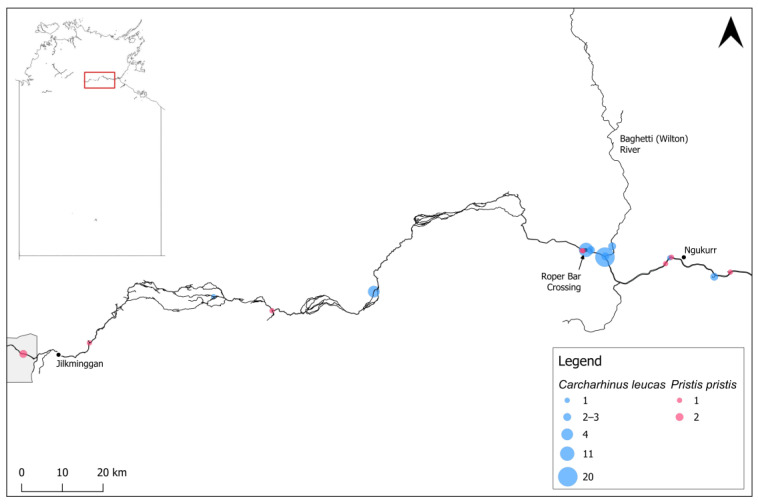
Species records in the upper Roper River, Northern Territory, Australia. Size of circles indicates the number of animals caught or observed in each location (refer to legend). All but one *Pristis pristis* locations are approximate based on catch locations described by members of the public. The grey shaded area represents Elsey National Park. *P. pristis* records from the Baghetti (Wilton) River outside the bounds of the map are not shown (~150 km from the Baghetti [Wilton]/Roper confluence) [17,36].

The most downstream capture location for *C. leucas* was at the Phelp and Roper confluence, approximately 34 km from the Roper River mouth. In 2023 surveys, *C. leucas* were not present downstream of the ‘Hawksnest’, a small rocky island approximately 80 km upstream from the mouth (Figure 2). *Carcharhinus leucas* also extend much further upstream into entirely fresh, clear water with substrates of rock, sand, and silt, with records up to ~300 km upstream (Figure 3).

### 3.2. Glaucostegus typus

Two *G. typus* visually estimated at ~100 cm TL were observed in November 2023, approximately 15 km upstream from the Roper River mouth (Figure 2). These were observed in <1 m depth, a water temperature of 30.5 °C, a turbidity of 19 NTU, and a salinity of 29.38 PSS. This species matures at 150–180 cm TL [37], indicating that these individuals were likely immature.

### 3.3. Glyphis glyphis

Seventy-eight neonate (n = 16) and immature (n = 62) *G. glyphis* were captured in the lower Roper River by hook-and-line over 494.3 hook hours in 2023 and 2024. Sharks ranged in size from 53.5 to 140.0 cm TL, with an average of 88.0 ± 22.4 cm TL. The sex ratio was 1:1 (F:M), without significant difference between the size of males and females (*t*-test, *p* = 0.45). Sixteen neonates were captured over the November 2023 and October 2024 surveys at 53.5–62.0 cm TL, while only larger juveniles were encountered in September 2023. Catches occurred in a range of salinities, from 0.58 to 20.8 PSS, and in turbidity of 12.2–549.0 NTU. Water depths ranged from 1.2 to 9.5 m and temperatures from 27.3 to 31.3 °C. CPUE was 0.16 individuals per hook hour.

*Glyphis glyphis* captures were restricted to a narrow estuarine stretch of the Roper River, with records from the Phelp River confluence to the northern end of Green Island, approximately 66 km from the river mouth (Figure 2). This section of the river has a substrate consisting primarily of mud and sand, resulting in higher turbidity. 

### 3.4. Pristis pristis

One immature male *P. pristis* was captured by gillnet in October 2017 at 2.4 m depth. This individual was 103.3 cm TL. No water quality data were recorded. CPUE was 0.004 individuals per 100 m net-hour.

The literature and data review produced a further ten *P. pristis* records, which were captured by baited hook-and-line, gillnet, or electrofished, or observed swimming by members of the public or other researchers in the Roper River and its tributaries during 2002–2024, ranging in size from ~100.0 to 340.0 cm TL [25,32,34,38,39]. A 103.0 cm TL male *P. pristis* was captured in May 2024 by Traditional Owners from the Baghetti (Wilton) River at the Baghetti Outstation, approximately 290 km upstream from the Roper River mouth and ~150 km from the Baghetti (Wilton)/Roper confluence [36]. *Pristis pristis* were also recorded as ‘common’ in a billabong nearby on the Baghetti (Wilton) River floodplain, where maximum depth was 2 m and Secchi depth was 10 cm, although specific records are not available [17]. The largest individual (340.0 cm TL) was a female captured at 5.0 m depth, a turbidity of 4 NTU, and an electrical conductivity of 1244.7 µS/cm, which equals <1.0 PSS (salinity) [39].

All *P. pristis* records occurred in fresh and generally very clear water with rocky/sandy/muddy substrates extending from ~80 km upstream from the Roper River mouth at the ‘Hawksnest’ to Elsey National Park near Mataranka (Figure 1), ~360 km from the mouth (Figure 2 and Figure 3). The largest recorded individual was captured at the furthest upstream site, at 12 Mile Yards Campground in Elsey National Park.

### 3.5. Urogymnus dalyensis

Fourteen *U. dalyensis* were captured in 2023 and 2024 by hook-and-line. Individuals were mature (n = 13) or subadult (n = 1), including four females, which were observed to be pregnant or possibly pregnant in September 2023, while one had possibly recently pupped (distended abdomen but not firm) in November. Animals ranged in size from 83.0 to 129.5 cm disc width (DW), with an average of 105.0 ± 12.3 cm DW. The sex ratio was 1:0.56 (F:M), with no significant difference between the size of males and females (*t*-test, *p* = 0.30). Total mass ranged from 16 kg to ~50 kg for the largest individual, a mature female. *Urogymnus dalyensis* were captured in depths of 2.0–7.0 m, salinities of 5.8–23.6 PSS, turbidity of 58–292 NTU, and temperatures of 27.0–30.5 °C.

The literature and data review produced one additional *U. dalyensis* record. In July 2002, an individual measuring 124.0 cm DW was caught in the lower Roper River at a salinity of 26.1 ppt, water temperature of 22.3 °C, depth of 5.0 m, and a Secchi depth of 30 cm [25]. *Urogymnus dalyensis* have also been recorded at Jilkminggan over several years (Yugul Mangi Rangers pers. obs.), although specific details are not available to incorporate individual records into this review. Records of this species therefore extend from the lower Roper River (~12 km from the mouth) (Figure 2) upstream to Jilkminggan (~350 km upstream from the mouth).

## 4. Discussion

The Roper River and its tributaries in the Northern Territory of Australia represent an important system for euryhaline elasmobranchs. Four of the five Australian euryhaline species have been recorded in the river, which is utilised as a reproductive area for all four species, indicated by the presence of early life stages and pregnant females. The lower estuarine stretch may also represent an important reproductive area for marine species, including the Critically Endangered giant guitarfish (*G. typus*) [40], which was recorded ~15 km upstream of the mouth in brackish water. Euryhaline elasmobranchs extend almost 400 km upstream throughout the Roper River, as well as almost 300 km into the Baghetti (Wilton) River. Sections of the Roper River’s mid-reaches are heavily braided, which is unique in northern Australia and caused by the area’s flat topography and sediment build-up behind choke points [27]. The stream morphology results in several sections of the river being too shallow for elasmobranchs to pass through, or they are cut off by dry sections aside from times of flood, limiting some up- and downstream movements to high flow events. The largest *P. pristis* was a female of 340 cm TL captured in Elsey National Park [39], which is significantly larger than all other records documented in this study. This species matures at 280–300 cm TL, at which time it generally returns to marine environments [41,42]. This may indicate that this individual had not been able to complete its downstream migration for several years due to a lack of river connectivity.

The sizes and life stages of the individuals recorded in the Roper River indicate that the system is a reproductive area for all four recorded euryhaline species. The sizes of *G. glyphis* neonates captured during this study fall within the expected size-at-birth range of 50–65 cm TL [22,43]. Neonate *C. leucas* ranged from 67.5 to 86 cm TL, with size-at-birth estimated at 67.5–78.5 cm TL in the Roper River (based on individuals with open umbilical scars only). Size-at-birth for *C. leucas* is understood to be 50–80 cm TL [44,45,46], with likely rapid growth in the first month, as the individuals larger than 80 cm TL recorded in this study had closed umbilical scars. Parturition likely occurs prior to the onset of the wet season for several species, with *C. leucas* and *G. glyphis* neonates captured in October and November, and pregnant *U. dalyensis* observed in September. While this is the first insight into reproductive seasonality for *U. dalyensis*, these results coincide with previously published results for *G. glyphis* (September–December) [22,47] and *C. leucas* (wet season in northern Australia) [48]. It should be noted that surveys were limited to the abovementioned months and therefore a full picture of seasonality is not available. No neonate or juvenile *U. dalyensis* were encountered and the habitat preferences of small individuals in the Roper River is unknown. Size-at-maturity for *U. dalyensis* is estimated at ~90 cm DW for males [37]. Males may mature at a slightly smaller size, with one male assessed as mature during this study at 87.0 cm DW, while a subadult was measured at 83.0 cm DW. No size-at-maturity is known for females [9]; however, the smallest female captured during this study was possibly pregnant at 95.0 cm DW. Our results also demonstrate a marginal increase to the maximum known size of *U. dalyensis* at 129.5 cm DW, exceeding the previously reported 124.0 cm DW (also from the Roper River) [25].

Targeted surveys resulted in a significant range extension for *G. glyphis*. Seventy-eight individuals ranging from neonate to ~4 years old based on the presence of visible umbilical scars and reported length-at-age data [49] were captured in the lower Roper River in 2023 and 2024. The presence of neonates and juveniles indicates that the river represents a reproductive area, as juveniles tend to remain in river systems for at least six years, or possibly until they mature at around 12 years old [24,49,50]. During these surveys, *G. glyphis* were recorded only from a restricted band of suitable habitat, which extended to ~66 km upstream of the mouth. Prior to this study, *G. glyphis* had only been considered extant in the Wenlock and Ducie Rivers in the Port Musgrave system of western Cape York, Queensland, throughout the Van Diemen Gulf in the NT, the western NT, and into the Kimberley region of Western Australia [22,51]. Preliminary data demonstrate connectivity between the Roper and Wenlock Rivers, based on a mature female that was tagged in the Wenlock River and travelled to the Roper River in the late dry season (September/October) [52], a straight-line distance of 805 km from the mouth of Port Musgrave (Wenlock River) to the mouth of the Roper River. Further research is required to develop a more thorough understanding of the Roper River population size, structure, and connectivity with the species’ wider range.

Recreational and commercial fishery activities in the Roper River have the potential to impact elasmobranch populations, including threatened species such as *G. glyphis* and *P. pristis*. Line fishing makes up 75% of recreational effort in the NT [53]. While many recreational fishers primarily use lures to target barramundi (*Lates calcarifer*), baited hook-and-line fishing regularly occurs, which results in increased elasmobranch bycatch [53,54]. Recreational fishers sometimes retain rather than release threatened elasmobranchs, and this has been seen with river sharks [55] and *P. pristis*, with an individual discarded on land at the McArthur River crossing, south of the Roper River [34]. Two commercial fisheries operate in the Roper River and its coastal waters: the NT Barramundi Fishery and the NT Mud Crab Fishery. The NT Barramundi Fishery utilises gillnets from 1 February to 30 September each year [30], resulting in river shark and sawfish bycatch and mortality [56]. The NT Mud Crab Fishery operating in the Roper River may capture neonate *G. glyphis* while they are still small enough to fit through the crab pot openings, as bycatch has been demonstrated in a similar fishery in Queensland [57]. In both fisheries, fishing gear may not be checked before captured individuals experience mortality [56,57] and threatened elasmobranchs may not be recorded or may be under-reported due to incorrect species identification [58].

Northern Australia is currently facing increased interest for water resource development for agriculture, industry, and mining [59,60]. The groundwater which ensures the Roper River continues to flow in deeper sections during the dry season [20] is the subject of great interest for water allocation licenses, including the Georgina Wiso Water Allocation, which recently granted the extraction of 210,000 megalitres per year from 2023 to 2031 [61]. Reduced availability of water as a result of anthropogenic water extraction will lead to an increased disruption of river connectivity, which has the potential to impact up- and downstream migrations of elasmobranchs [62,63], and anthropogenic effects may be compounded by reduced rainfall driven by climate change [64,65]. Sawfish are recognised as one of the species most likely to be significantly impacted by the alteration of flow regimes [27]; however, the effects of flow alteration have not been explored for other sharks or rays in the Roper River. This study does not provide any insights into how water extraction may affect habitat and therefore habitat use by euryhaline species. As an important reproductive area for threatened species, a thorough understanding of critical habitat use is crucial for species management. Further research is needed to develop an understanding of euryhaline species movements in the Roper River (in flood and drought events), as well as a detailed understanding of abundance, population size, and connectivity. The *G. glyphis* population in the Roper River, for example, is unlikely to be replaced by individuals from other rivers if it underwent a decline, due to reproductive philopatry in females [23,66].

## 5. Conclusions

Northern Australia is considered a ‘lifeboat’ region for threatened elasmobranchs [10,11,12]; however, many areas are understudied. The Roper River and its tributaries represent an important system for euryhaline elasmobranchs, with species extending from the mouth to almost 400 km upstream. The Roper River is a reproductive area for the threatened *G. glyphis*, representing a significant geographic range extension for the species. This study provides insights into parturition seasonality for all four observed euryhaline species, as well as new insights into life history for *U. dalyensis*. This is also the first study to map the spatial distribution of euryhaline elasmobranch records in the Roper River. Some of the records presented here were provided by members of the public and highlight the valuable contribution to knowledge of threatened species in extremely remote areas like the Roper River that citizen scientists can provide. The temporal constraints of the survey periods and records compiled here may have affected species catch rates due to interannual variability in recruitment and distribution. This is beyond the scope of this study, and a more thorough survey program would be required to understand this variability. Indeed, further research on this system is required to gain additional insights into life history and ecology, habitat requirements and use, and connectivity with other populations, particularly for threatened species. Critically, further research is required to understand the potential impacts of human-driven changes to habitats and therefore life history, as well as to improve the monitoring of fisheries interactions.

## Figures and Tables

**Table 1:** Summary of shark and ray records from the Roper River and its tributaries in the Northern Territory, Australia (excluding coastal waters). Records are from both the targeted surveys and the literature and data review. Size ranges are total length (TL) for all species except for *U. dalyensis*, for which disc width (DW) is reported. Size and mean size include data from the literature where size was reported. Categories are reported for the IUCN Red List of Threatened Species [6] and the Australian *Environment Protection and Biodiversity Conservation Act* 1999 (‘*EPBC Act*’) [35]. The numbers of individuals caught during surveys and derived from the literature are provided in brackets: (surveys, literature). *Glaucostegus typus* and *Glyphis glyphis* were recorded only during surveys. LC, Least Concern; VU, Vulnerable; CR, Critically Endangered; nl, not listed; std. dev., standard deviation. * indicates an approximate length.

Species	IUCN Red List/*EPBC Act* Category	Number Recorded	Size (cm)	Mean Size ± Std. Dev. (cm)	Depth (m)	Salinity (PSS)	Turbidity (NTU)
*Carcharhinus leucas*	VU/nl	83 (69, 14)	67.5–139.5	83.4 ± 16.0	0.8–21.0	0.06–11.9	3.2–529.0
*Glaucostegus typus*	CR/nl	2	~100.0 *	—	<1.0	29.38	19.0
*Glyphis glyphis*	VU/CR	78	53.5–140.0	88.0 ± 22.4	1.2–9.5	0.58–20.8	12.2–549.0
*Pristis pristis*	CR/VU	11 (1, 10)	100.0–340.0	135.3 ± 142.3	<2.0–5.0	<1.0	4.0
*Urogymnus dalyensis*	LC/nl	15 (14, 1)	83.0–129.5	106.2 ± 12.9	2.0–7.0	5.8–26.1	8.0–292.0

## Data Availability

Data are available in the Supplementary Materials. More detailed data may be requested from the corresponding author by email.

## Supplementary Materials

The following supporting information can be downloaded at: https://www.mdpi.com/article/10.3390/ani14223306/s1, Table S1: Elasmobranchs captured during dedicated surveys of the Roper River in the Northern Territory, Australia, between 2016 and 2024; Table S2: Elasmobranch records from the Roper River, Northern Territory, Australia, compiled from the literature and data review.
