# Supplementary material for: Sharks and Rays of Northern Australia’s Roper River, with a Range Extension for the Threatened Speartooth Shark Glyphis glyphis
Source: Animals (Basel). 2024 Nov 17;14(22):3306. doi: 10.3390/ani14223306 (PMC11590893; doi:10.3390/ani14223306)
Supplement: Supplementary file 1 [file animals-14-03306-s001.zip › animals-3239485-supplementary.pdf]

Supplementary Information

Julia M. Constance, Erica A. Garcia, Yugul Mangi Rangers, Christy-Louise Davies, Peter M. Kyne

Table S1. Elasmobranchs captured during dedicated surveys of the Roper River, Northern Territory, Australia between 2016 and 2024.
TL, total length; DW, disc width; TM, total mass; N, neonate; J, juvenile; S, subadult; M, mature.

Species	Date	Latitude	Longitude	Sex	Maturity	TL (cm)
Carcharhinus leucas	31/10/16	-14.712	134.511	♂	N	69.5
	31/10/16	-14.712	134.511	♀	N	73.2
	31/10/16	-14.712	134.511	♀	J	139.5
	31/10/16	-14.712	134.511	♀	N	72.8
	1/11/16	-14.729	134.551	♂	N	73.0
	1/11/16	-14.729	134.551	♂	N	72.2
	1/11/16	-14.729	134.551	♂	N	74.5
	1/11/16	-14.729	134.551	♂	N	73.6
	1/11/16	-14.729	134.551	♂	N	74.1
	1/11/16	-14.729	134.551	♂	J	122.2
	1/11/16	-14.729	134.551	♀	N	75.8
	1/11/16	-14.729	134.551	♀	N	78.5
	1/11/16	-14.729	134.551	♀	J	133.0
	1/11/16	-14.729	134.551	♀	N	73.2
	1/11/16	-14.729	134.551	♂	N	74.5
	1/11/16	-14.729	134.551	♂	J	111.0
	1/11/16	-14.727	134.553	♀	J	124.5
	1/11/16	-14.727	134.553	♂	N	74.5
	1/11/16	-14.730	134.553	♀	N	68.3
	1/11/16	-14.730	134.553	♂	N	76.0
	1/11/16	-14.730	134.553	♂	N	73.3
	1/11/16	-14.730	134.553	♀	N	73.0
	1/11/16	-14.730	134.553	♂	N	69.0
	1/11/16	-14.730	134.553	♂	N	82.3
	1/11/16	-14.730	134.553	♂	N	68.0
	2/11/16	-14.704	134.569	♀	N	68.5
	2/11/16	-14.704	134.569	♀	N	77.0
	2/11/16	-14.704	134.569	♂	N	78.5
	17/10/17	-14.713	134.509	♂	J	135.5
	18/10/17	-14.803	134.010	♂	N	77.0
	18/10/17	-14.803	134.010	♀	N	86.0
	18/10/17	-14.803	134.010	♂	N	78.0
	18/10/17	-14.803	134.010	♀	J	85.5
	20/10/17	-14.711	134.520	♀	J	115.0
	26/09/23	-14.820	135.037	♀	J	78.5
	26/09/23	-14.820	135.037	♀	J	81.0
	8/10/24	-14.783	135.116	♂	J	80.0
	8/10/24	-14.783	135.116	♂	J	79.5
	8/10/24	-14.783	135.116	♂	J	83.5
	8/10/24	-14.783	135.116	♂	J	83.5
	8/10/24	-14.783	135.116	♀	J	80.0
	8/10/24	-14.783	135.116	♂	J	79.5

Species	Date	Latitude	Longitude	Sex	Maturity	TL (cm)
	8/10/24	-14.783	135.116	♀	J	78.5
	8/10/24	-14.783	135.116	♀	J	82.0
	8/10/24	-14.783	135.116	♂	J	83.0
	8/10/24	-14.783	135.116	♂	J	80.0
	8/10/24	-14.783	135.116	♀	J	80.5
	9/10/24	-14.783	135.117	♀	N	82.0
	9/10/24	-14.783	135.117	♂	J	67.5
	9/10/24	-14.783	135.117	♂	J	79.0
	9/10/24	-14.680	135.271	♂	J	103.0
	10/10/24	-14.769	135.159	♀	N	76.5
	10/10/24	-14.769	135.159	♂	J	84.5
	12/10/24	-14.783	135.116	♀	J	82.0
	12/10/24	-14.783	135.117	♂	J	78.5
	12/10/24	-14.783	135.117	♂	J	73.5
	12/10/24	-14.783	135.117	♀	J	78.5
	12/10/24	-14.783	135.117	♀	J	80.0
	12/10/24	-14.783	135.117	♂	J	75.0
	12/10/24	-14.783	135.117	♀	J	83.0
	12/10/24	-14.783	135.117	♂	J	77.0
	12/10/24	-14.783	135.117	♂	J	90.0
	12/10/24	-14.783	135.117	♂	J	86.0
	12/10/24	-14.783	135.117	♀	J	88.0
	12/10/24	-14.783	135.117	♀	J	80.0
	12/10/24	-14.783	135.117	♂	J	103.0
	13/10/24	-14.783	135.117	♀	J	82.0
	13/10/24	-14.783	135.117	♀	J	80.0
	13/10/24	-14.783	135.117	♂	J	75.0
Pristis pristis	19/10/17	-14.715	134.502	♂	J	103.3
Glyphis glyphis	22/09/23	-14.683	135.262	♂	J	125.0
	23/09/23	-14.772	135.114	♂	J	79.5
	23/09/23	-14.772	135.114	♀	J	67.0
	23/09/23	-14.772	135.114	♀	J	65.0
	23/09/23	-14.772	135.114	♀	J	76.0
	23/09/23	-14.743	135.139	♀	J	79.0
	24/09/23	-14.765	135.155	♂	J	76.0
	1/11/23	-14.680	135.271	♂	N	54.7
	3/11/23	-14.783	135.117	♀	N	56.0
	3/11/23	-14.783	135.117	♂	J	111.0
	3/11/23	-14.783	135.117	♂	J	107.0
	3/11/23	-14.783	135.117	♂	J	77.5
	3/11/23	-14.783	135.116	♂	J	119.0
	3/11/23	-14.783	135.116	♂	J	77.0
	3/11/23	-14.783	135.116	♀	J	76.0
	3/11/23	-14.783	135.116	♂	J	83.5
	4/11/23	-14.783	135.117	♂	N	62.0
	4/11/23	-14.783	135.117	♂	N	55.5
	4/11/23	-14.783	135.117	♀	J	82.5
	4/11/23	-14.783	135.117	♂	J	90.5

Species	Date	Latitude	Longitude	Sex	Maturity	TL (cm)
	4/11/23	-14.783	135.117	♂	J	111.0
	4/11/23	-14.783	135.117	♀	J	117.0
	4/11/23	-14.783	135.117	♂	J	77.5
	4/11/23	-14.783	135.117	♀	J	111.0
	4/11/23	-14.783	135.117	♀	J	109.0
	4/11/23	-14.783	135.117	♀	N	53.5
	4/11/23	-14.783	135.116	♀	J	83.0
	5/11/23	-14.783	135.117	♂	N	62.0
	5/11/23	-14.783	135.117	♂	N	62.0
	5/11/23	-14.783	135.117	♂	J	76.5
	5/11/23	-14.783	135.117	♂	J	82.0
	5/11/23	-14.783	135.117	♀	N	62.0
	5/11/23	-14.783	135.117	♂	J	80.0
	5/11/23	-14.783	135.117	♀	J	88.5
	5/11/23	-14.783	135.117	♀	N	55.5
	8/11/23	-14.783	135.117	♂	J	105.0
	8/11/23	-14.783	135.117	♀	J	114.0
	8/11/23	-14.783	135.117	♀	J	105.0
	8/11/23	-14.783	135.117	♂	J	85.0
	8/11/23	-14.783	135.117	♀	J	72.0
	8/10/24	-14.783	135.116	♀	J	117.5
	8/10/24	-14.783	135.116	♀	J	86.5
	8/10/24	-14.783	135.116	♀	J	72.0
	8/10/24	-14.783	135.116	♂	J	78.5
	8/10/24	-14.783	135.116	♀	J	105.0
	8/10/24	-14.783	135.116	♂	J	105.0
	8/10/24	-14.783	135.116	♀	J	103.5
	8/10/24	-14.783	135.116	♂	J	97.0
	8/10/24	-14.783	135.116	♀	J	93.0
	8/10/24	-14.783	135.116	♂	J	108.5
	8/10/24	-14.783	135.116	♀	J	77.0
	8/10/24	-14.783	135.116	♀	J	98.0
	8/10/24	-14.783	135.116	♀	J	79.5
	8/10/24	-14.783	135.116	♀	J	97.0
	8/10/24	-14.783	135.116	♂	J	79.0
	9/10/24	-14.680	135.271	♂	J	86.5
	9/10/24	-14.680	135.271	♂	J	126.5
	9/10/24	-14.680	135.271	♂	J	103.5
	10/10/24	-14.680	135.271	♀	J	103.5
	10/10/24	-14.680	135.271	♂	J	77.0
	11/10/24	-14.679	135.271	♂	N	56.0
	11/10/24	-14.679	135.271	♂	J	81.5
	11/10/24	-14.679	135.271	♂	N	61.0
	11/10/24	-14.679	135.271	♂	N	58.0
	11/10/24	-14.679	135.271	♀	N	58.5
	11/10/24	-14.679	135.271	♂	J	105.5
	12/10/24	-14.783	135.116	♂	J	109.0
	12/10/24	-14.783	135.117	♀	J	136.5

Species	Date	Latitude	Longitude	Sex	Maturity	TL (cm)	
	12/10/24	-14.783	135.117	♂	J	111.0	
	12/10/24	-14.783	135.117	♀	J	122.5	
	12/10/24	-14.783	135.117	♀	J	80.0	
	12/10/24	-14.783	135.117	♀	J	61.5	
	12/10/24	-14.783	135.117	♀	J	109.0	
	13/10/24	-14.783	135.117	♂	N	55.5	
	13/10/24	-14.783	135.117	♀	J	118.5	
	13/10/24	-14.783	135.117	♀	J	120.0	
	13/10/24	-14.783	135.117	♀	J	140.0	
	13/10/24	-14.783	135.117	♀	N	58.5	
**Species**	**Date**	**Latitude**	**Longitude**	**Sex**	**Maturity**	**DW (cm)**	**TM (kg)**
Urogymnus dalyensis	22/09/23	-14.681	135.271	♂	M	112.0	33.0
	22/09/23	-14.683	135.262	♂	M	100.0	31.0
	23/09/23	-14.772	135.114	♀	M	87.0	20.5
	24/09/23	-14.765	135.155	♀	M	112.0	34.0
	26/09/23	-14.684	135.262	♂	M	110.0	34.0
	26/09/23	-14.684	135.262	♀	M	95.0	25.5
	27/09/23	-14.661	135.269	♀	M	98.0	26.0
	27/09/23	-14.661	135.269	♂	S	83.0	16.0
	1/11/23	-14.680	135.271	♀	M	129.5	50.0
	2/11/23	-14.683	135.262	♂		96.0	25.0
	2/11/23	-14.765	135.155	♀		112.5	36.0
	2/11/23	-14.765	135.155	♀	M	113.0	38.0
	2/11/23	-14.684	135.259	♀		111.0	33.0
	9/10/24	-14.680	135.271	♀		111.0	41.0

Table S2. Elasmobranch records from the Roper River, Northern Territory, Australia compiled from the literature and data review.
TL, total length; DW, disc width; DITT, Department of Industry, Tourism and Trade; DEPWS, Department of Environment, Parks and Water Security.

Species	Date	Latitude	Longitude	Sex	TL (cm)	DW (cm)	No.	Source
Carcharhinus leucas	07/02	-14.730	134.551				1	Thorburn *et al.*, 2003
	07/02	-14.713	134.519				2	Thorburn *et al.*, 2003
	07/02	-14.713	134.508				6	Thorburn *et al.*, 2003
	07/02	-14.774	134.802				3	Thorburn *et al.*, 2003
	25/09/08	-14.820	133.656				1	Dally & Larson, 2008
	2/05/21	-14.7	134.7	♀			1	ALA, 2024
Pristis pristis	27/09/79	-13.758	134.466				>1	Midgley, 1979
	07/02	-14.927	133.372				1	Thorburn *et al.*, 2003

**Species**	**Date**	**Latitude**	**Longitude**	**Sex**	**TL (cm)**	**DW (cm)**	**No.**	**Source**
	17/09/17	-14.951	133.220				1	S. Irvine, pers. comm.
	15/05/18	-14.744	134.691				1	NT DITT, unpubl. data
	19/10/19	-14.71	134.5	♂			1	ALA, 2024
	15/05/21	-14.819	135.037		100.0		1	NT DITT, unpubl. data
	21/09/21	-14.951	133.220	♀	3400.0		1	DEPWS, 2022
	21/10/21	-14.729	134.704		109.0		1	NT DITT, unpubl. data
	12/11/22	-14.852	133.790				1	NT DITT, unpubl. data
	3/04/23	-14.764	134.839		87.0		1	C. Perna, pers. comm., 2023
	05/24	-13.759	134.463	♂	103.0		1	S. Miller, pers. comm., 2024
Urogymnus dalyensis	07/02	-14.727	135.322			124.0	1	Thorburn *et al.*, 2003
		-14.952	133.301				>1	Yugul Mangi Rangers pers. obs.
